# Optical coherence tomography angiography measurements in multiple sclerosis: a systematic review and meta-analysis

**DOI:** 10.1186/s12974-023-02763-4

**Published:** 2023-03-27

**Authors:** Soheil Mohammadi, Mahdi Gouravani, Mohammad Amin Salehi, J. Fernando Arevalo, Steven L. Galetta, Hamid Harandi, Elliot M. Frohman, Teresa C. Frohman, Shiv Saidha, Neda Sattarnezhad, Friedemann Paul

**Affiliations:** 1 School of Medicine, Tehran University of Medical Sciences, Pour Sina St, Keshavarz Blvd, Tehran, 1417613151 Iran; 2 Wilmer Eye Institute, Johns Hopkins University School of Medicine, Baltimore, USA; 3 Department of Neurology, New York University Langone Medical Center, New York, NY USA; 4 Laboratory of Neuroimmunology, Stanford University School of Medicine, Stanford, CA USA; 5 Division of Neuroimmunology and Neurological Infections, Department of Neurology, Johns Hopkins University, Baltimore, MD USA; 6 Division of Multiple Sclerosis and Neuroimmunology, Department of Neurology, Stanford Multiple Sclerosis Center, Stanford University, Stanford, USA; 7 Department of Neurology, Experimental and Clinical Research Center, Max Delbrueck Center for Molecular Medicine, NeuroCure Clinical Research Center, Charité Universitätsmedizin Berlin, Berlin, Germany

**Keywords:** Multiple sclerosis, MS, Optical coherence tomography, Optical coherence tomography angiography, OCT, OCT-A, Retina, Perfusion density, Vascular density, Meta-analysis

## Abstract

**Background and objectives:**

Recent literature on multiple sclerosis (MS) demonstrates the growing implementation of optical coherence tomography–angiography (OCT-A) to discover potential qualitative and quantitative changes in the retina and optic nerve. In this review, we analyze OCT-A studies in patients with MS and examine its utility as a surrogate or precursor to changes in central nervous system tissue.

**Methods:**

PubMed and EMBASE were systematically searched to identify articles that applied OCT-A to evaluate the retinal microvasculature measurements in patients with MS. Quantitative data synthesis was performed on all measurements which were evaluated in at least two unique studies with the same OCT-A devices, software, and study population compared to controls. A fixed-effects or random-effects model was applied for the meta-analysis based on the heterogeneity level.

**Results:**

The study selection process yielded the inclusion of 18 studies with a total of 1552 evaluated eyes in 673 MS-associated optic neuritis (MSON) eyes, 741 MS without optic neuritis (MSNON eyes), and 138 eyes without specification for the presence of optic neuritis (ON) in addition to 1107 healthy control (HC) eyes. Results indicated that MS cases had significantly decreased whole image superficial capillary plexus (SCP) vessel density when compared to healthy control subjects in the analyses conducted on Optovue and Topcon studies (both *P* < 0.0001). Likewise, the whole image vessel densities of deep capillary plexus (DCP) and radial peripapillary capillary (RPC) were significantly lower in MS cases compared to HC (all* P* < 0.05). Regarding optic disc area quadrants, MSON eyes had significantly decreased mean RPC vessel density compared to MSNON eyes in all quadrants except for the inferior (all* P* < 0.05). Results of the analysis of studies that used prototype Axsun machine revealed that MSON and MSNON eyes both had significantly lower ONH flow index compared to HC (both *P* < 0.0001).

**Conclusions:**

This systematic review and meta-analysis of the studies reporting OCT-A measurements of people with MS confirmed the tendency of MS eyes to exhibit reduced vessel density in the macular and optic disc areas, mainly in SCP, DCP, and RPC vessel densities.

**Supplementary Information:**

The online version contains supplementary material available at 10.1186/s12974-023-02763-4.

## Introduction

Multiple sclerosis (MS) is an inflammatory auto-immune disease causing demyelination and axonal loss in the central nervous system (CNS) with varying geographical prevalence and incidence rates [1, 2]. The presence of myelin-reactive T cells in MS plaques and the effective response of MS patients to immunomodulatory drugs targeting these cells supports the theory that MS primarily results from dysregulation in the cellular immune system [3]. Aside from immune-based etiologies, there is now growing attention to discovering possible metabolic and vascular elements contributing to the pathogenesis of this disorder [4]. Because of a higher incidence among young adults, this condition can result in considerable disability for affected individuals [5, 6]. Therefore, the importance of finding clinically useful, non-invasive, and objective biomarkers to detect MS in the early stages is important.

The eye offers one gateway to studying the CNS tissue. The retina and brain share the same diencephalic origin and have analogous neuronal layers, including a ganglion cell layer (GCL), a retinal nerve fiber layer (RNFL) in addition to a vascular supply [7, 8]. Thus, the retina can be highly influenced by pathologies, such as Alzheimer’s disease, migraine, MS, neuromyelitis optica spectrum disorder (NMOSD), and myelin oligodendrocyte glycoprotein-associated disease (MOGAD), which are primarily known to also involve other parts of the CNS [9–12]. Optic neuritis (ON), characterized by acute visual loss and eye pain, is a common manifestation of MS [13, 14]. Approximately half of MS patients experience ON during the course of their disease [15].

The location of the eye makes the retina a highly accessible structure for non-invasive imaging, and it may serve as a surrogate for brain pathology [8]. Optical coherence tomography (OCT), which uses low-coherence light to capture cross-sectional and high-resolution images from the retinal and choroidal layers, has been broadly employed [16].

Application of OCT to examine the retinal layers in MS-associated optic neuritis (MSON) and MS without optic neuritis (MSNON) has revealed thinning and neurodegeneration in the peripapillary retinal nerve fiber layer (pRNFL), macular ganglion cell layer and inner plexiform layer (GCIPL) of the patients compared to the healthy controls [10]. Furthermore, investigating the role of vascular abnormalities in the establishment and progression of MS lesions is gaining popularity. These abnormalities can stem from cerebral endothelial cell dysfunction and may lead to hypoperfusion and hypoxia of CNS tissue [17]. Likewise, the anterior visual pathway requires a high blood flow rate to meet the supply and demand characteristics of this highly active metabolic system [18]. A recent study showed an abnormal retinal microcirculation in patients with relapsing–remitting multiple sclerosis (RRMS) [19]. In addition, blood flow in the ocular vascular system may be diminished in association with ON, potentially resulting in impaired visual acuity [20].

As a promising novel imaging technique, OCT–angiography (OCT-A) provides in-vivo depth-resolved images of the retinal and choroidal microvasculature [21]. The results of a meta-analysis on the application of OCT-A in dementia revealed a significant increase in the foveal avascular zone (FAZ) area in patients with Alzheimer’s disease [9].

To date, a considerable number of studies have examined retinal vasculature in MS patients using different OCT-A devices and methods, unveiling significant changes compared to healthy controls (HC) and correlations with several variables including disability scores, highlighting the promising capability of this technology to improve our current understanding of this disorder [8, 22]. MS, NMOSD, and MOGAD, which have different prognoses and treatments, may have overlapping presentations, such as ON, making it difficult to distinguish them in the acute clinical setting [23–26]. Qualitative differences in retinal microvasculature captured by OCT-A together with regular OCT may help to mitigate the challenge in the differentiation of these disorders [27–30]. Correlations have been noted between OCT-A measurements of people with MS and their level of disability assessed by expanded disability status scale (EDSS) score and visual outcome measures [22, 31].

Despite the broad interest in applying OCT-A technology in people with MS, there remain important issues regarding image quality, the development of uniform standards and methodologies, and the interpretation of resultant images [32, 33]. In this systematic review and meta-analysis, we attempted to accumulate the findings and quantitively compare the results, where possible, between the studies that used OCT-A in people with MS to find possible agreements and inconsistencies among the various OCT-A methodologies.

## Methods

The current systematic review and meta-analysis was conducted according to evidence-based criteria provided by the Preferred Reporting Items for Systematic Reviews and Meta-Analyses (PRISMA) guideline [34]. The reviewers submitted the developed study protocol to the International Prospective Register of Systematic Reviews (PROSPERO) website with the Registration Number CRD42021275881.

### Search strategy

PubMed and EMBASE were systematically searched to identify relevant articles from the earliest published record until May 2021 (The search was updated on May 15, 2022). The following combination of key terms was used to form the search strategy for each database: (“optical coherence tomography angiography” OR “OCT angiography” OR “OCTA” OR “optical coherence tomographic angiography”) AND (“multiple sclerosis” OR “MS” OR “Sclerosis, Disseminated” OR “Disseminated Sclerosis” OR “Multiple Sclerosis, Acute Fulminating”). There was no filter regarding publication location, age group of participants, and type of MS in the search process. To minimize the risk of missing eligible studies, we also performed a manual search by screening the references of included studies.

### Eligibility criteria

Published studies that applied OCT-A to evaluate the retinal microvasculature measurements in patients with any type of MS and MSON were included in this review if able to fulfill the following conditions: (a) written in English; (b) original peer-reviewed studies; (c) diagnosis of any type of MS and MS-associated ON were confirmed according to participants’ medical records or through established criteria, such as the 2005, 2010, and 2017 McDonald Criteria [35]; (d) presence of control group. Studies with the following characteristics were excluded: (a) non-English; (b) non-original; (c) non-human; (d) case reports, reviews, book chapters, letters, and conference abstracts; (e) lack of a control group; (f) studies not applying OCT-A.

### Data extraction

After the primary and detailed screening of retrieved unique articles, the following data were extracted from the included studies by two independent authors (MAS and MG): (1) first author and publication year; (2) utilized diagnostic criteria for MS and ON; (3) EDSS score and other taken additional scoring exams; (4) number, trait, mean age, male percentage and selection criteria of participants in each case and control groups; (5) disease duration; (6) best corrected visual acuity (LogMAR) and intraocular pressure (IOP) of enrolled eyes; (7) type of OCT-A and image analysis software applied; (8) OCT-A measurements; (9) observed alterations in OCT-A metrics including superficial, deep, and radial peripapillary capillary (RPC), macula, optic disc, and choriocapillaris layer vascular densities, FAZ area, and blood flow velocity. Any disagreements in the data extraction process were arbitrated by the third author (SM).

### Statistical analysis

Quantitative data synthesis was performed on all OCT-A measurements of MS patients reported in mean ± SD format, which were evaluated in at least two unique studies with the same OCT-A devices and image analysis software and the same study population (MSON, MSNON, and MS) compared to controls. Stata version 16 software (StataCorp, College Station, TX) was used to conduct all meta-analysis, and effect sizes of difference between case and control groups were reported as the mean difference (MD) with a confidence interval (CI) of 95% and a *P* value less than 0.05 was considered as statistically significant. The heterogeneity across the studies was calculated using Higgin’s *I*^2^ test, for which a level of heterogeneity of less than 40% is considered unimportant according to the Cochrane manual, and the fixed-effects model was used to carry out analysis in this case. However, if *I*^2^ was higher than 40%, the random-effects approach was applied.

### Metrics and terminology

**Fig. 1:**
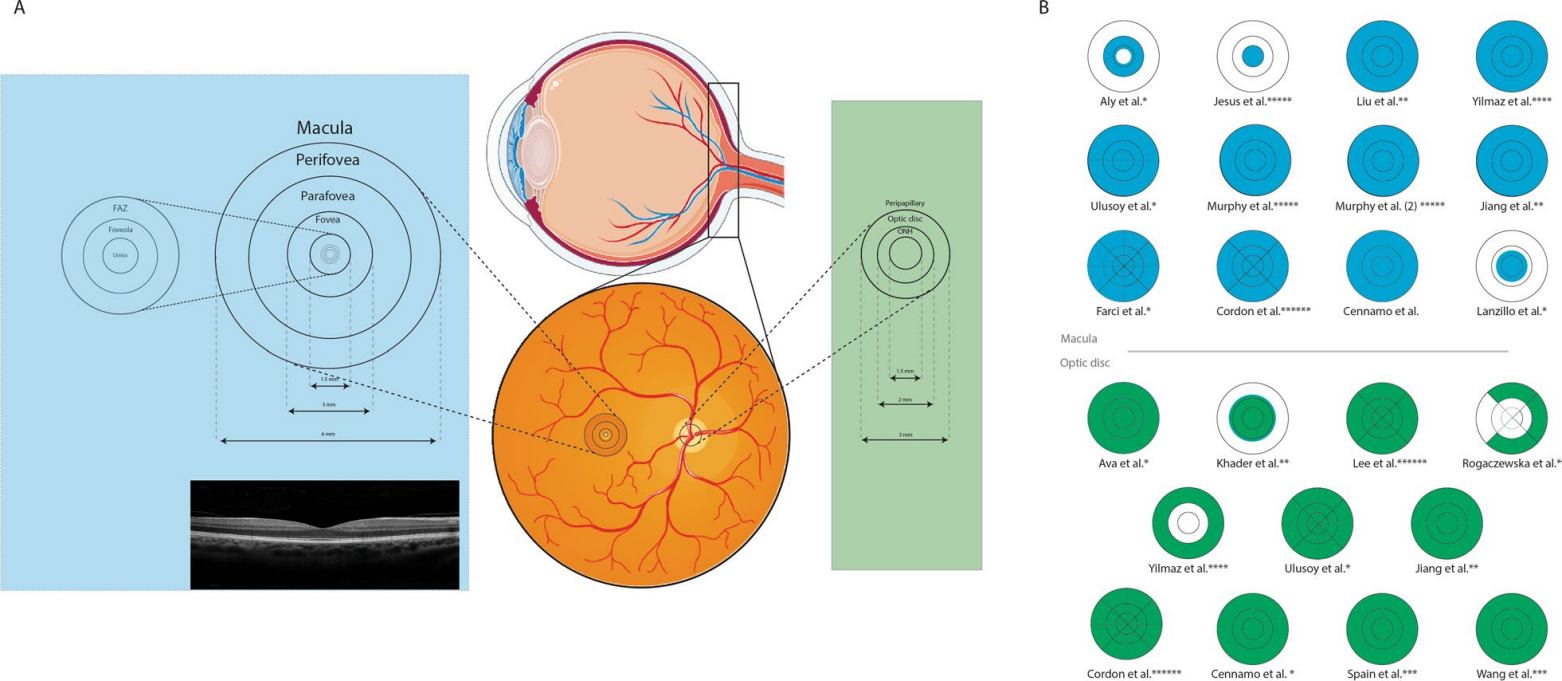
Macula and optic disc regions of each included study which were measured for vascular, perfusion, and flow density. **A** Illustration of retinal fundus and location of the macula and optic disc segments. Foveal avascular zone (FAZ) parts are also displayed in the left circle. **B** Fields analyzed in each study. Blue shows sections of macula and green shows sections of the optic disc. * indicates studies using an Optovue machine, ** Indicates studies using a Zeiss machine. *** indicates studies using a prototype Axsun SS-OCT machine, **** indicates studies using a Nidek machine, ***** indicates studies using a Heidelberg machine, and ****** indicates studies using a Topcon machine. Note: Parts of the figure were drawn using pictures from Servier Medical Art. Servier Medical Art by Servier is licensed under a Creative Commons Attribution 3.0 Unported License (https://creativecommons.org/licenses/by/3.0/)

As there was a considerable difference between applied OCT-A devices and software leading to discrepancies in segmentation boundaries for the measured retinal layers in the included studies, we only compared the results which have been reported by at least two studies with the same OCT-A models and analysis software. Additional file 1: Table S1 comprehensively presents the measurement terminology used by each included study and their definitions provided in the corresponding manuscript. Since there are differences in the nomenclature used by the articles for the measured layers, for the sake of consistency in the current review, we referred to the superficial (retinal) capillary plexus (layer) as the “superficial capillary plexus” (SCP) and the deep (retinal) capillary plexus (layer) as “deep capillary plexus” (DCP). The study by Khader et al. defined an outer retina layer stretching from the outer boundary of the outer plexiform layer to an end at Bruch’s membrane lever [36]. However, it should be noted that diverse boundary definitions may be provided by each study for these layers, thus limiting the comparability of the results (Additional file 2: Table S2; Fig. 1).

As the most conducted measurement in studies, vessel density defined as the proportion of vessels with blood flow was reported in three distinct formats: (1) length-based (the total length of the perfused vasculature per measured unit area); (2) area-based (the total area of the perfused vasculature per unit area); (3) volume-based (the vessel density per volume unit in the same area). Length-based measurements are supposed to detect alterations in smaller capillaries more sensitively as they are less prone to bias caused by the larger vasculature of the retina [37].

Optovue studies reported two metrics that were: (1) FAZ area; (2) area-based density metric referred to as vascular density, vessel density, or flow density that in this review will collectively be referred to as “vessel density”.

Four metrics reported by the studies with Zeiss device were: (1) FAZ area; (2) area-based perfusion density; (3) vessel density metric presented in two forms including a length-based vascular density and a vessel density based on monofractal analysis (Dbox) that in this review will collectively be referred to as “vessel density”; (4) volumetric vessel density defined as the vessel density (as Dbox) divided by the corresponding tissue volume in the same area [38].

The study with the Nidek device reported four metrics including: (1) FAZ area; (2) FAZ perimeter; (3) FAZ circularity index (indicating the degree of resemblance of the area to a perfect circle for which values closer to one means a higher circularity); (4) an area-based vascular density that in this review will be referred to as “vessel density”.

Studies with the Heidelberg device only reported an area-based vessel density. Two studies with Topcon device also reported an area-based vascular density that in this review will be referred to as “vessel density”. The only reported measurement in the studies with prototype Axsun device was flow index. The flow index is defined as the mean value of the flow signal in an en-face OCT-A image. Flow index has been speculated to be more sensitive to changes in velocity or volumetric flow than vessel density; however, it is not as valid for disease diagnosis due to an abundant dependence on the strength of the OCT-A reflectance signal [39].

As listed in Additional file 2: Table S2, various areas and subsectors of the superficial and deep macula (whole image, fovea, parafovea, and perifovea as well as superior, inferior, temporal, and nasal quadrants, and superior and inferior hemispheres) and RPC and superficial layers of the optic disc were measured by OCT-A studies, however, with different fields of scan and defined boundaries making it unfeasible to compare the results across the studies. The Early Treatment Diabetic Retinopathy Study (ETDRS) grid used in OCT scans divides the macula into three concentric circles, including the central circle (1 mm in diameter), inner macular ring (3 mm in diameter), and outer macular ring (6 mm in diameter), which, in this review, are referred to as the fovea, parafovea, and perifovea, respectively [40]. Parafoveal and perifoveal areas may then split into four quadrants: superior, inferior, temporal, and nasal.

## Results

**Fig. 2:**
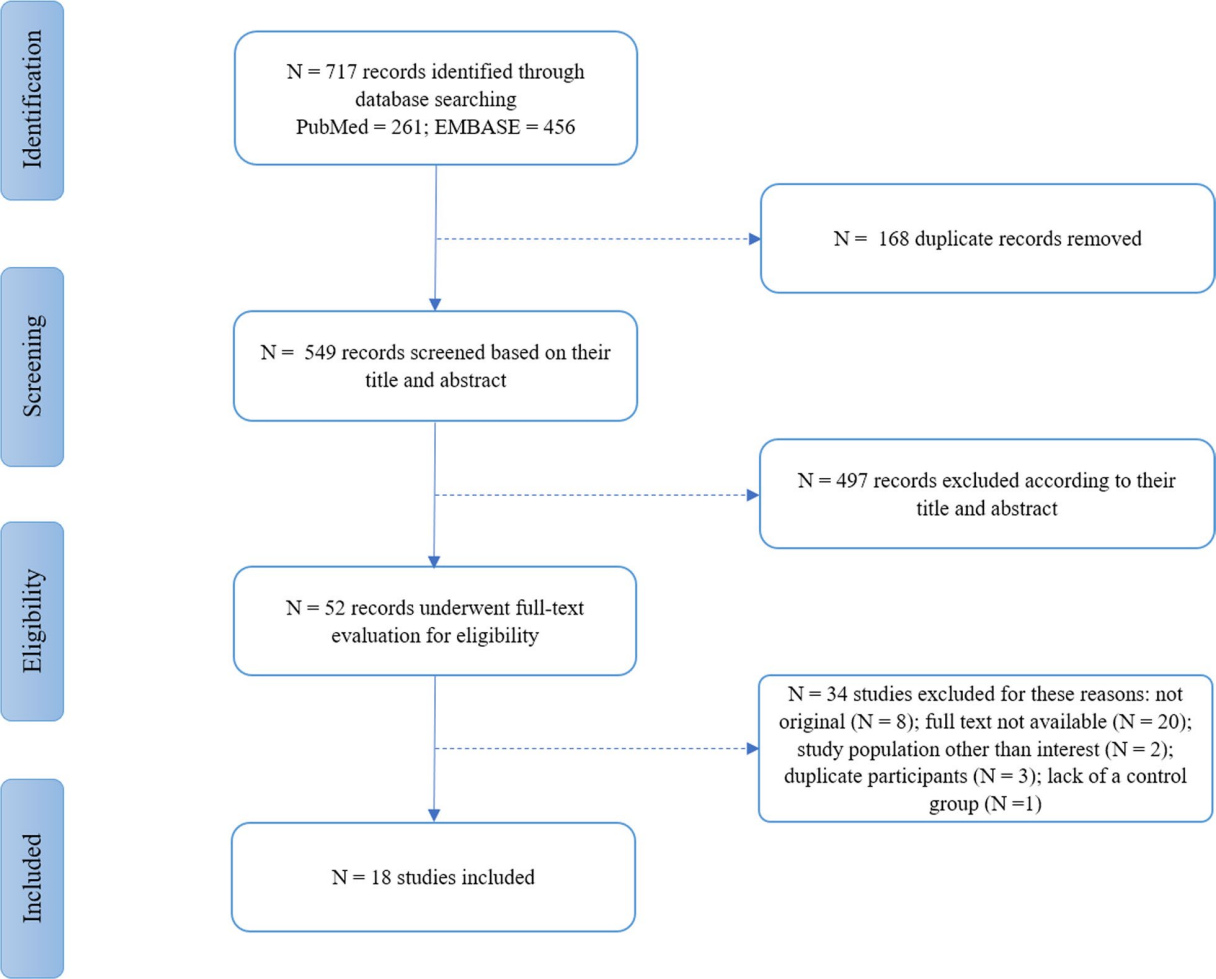
Flow diagram of study selection process

The search of electronic databases resulted in the identification of 717 records, of which 168 articles were duplicates and were removed. The remaining underwent screening based on title and abstract, which led to the exclusion of 497 irrelevant records. Finally, 52 articles were thoroughly evaluated to include eligible studies. In this phase, 14 studies were omitted due to the unavailability of full-text manuscripts. Twelve studies were excluded, because they were not original. Two studies were removed, since their cases were a combination of people with MS as well as a clinically isolated syndrome (CIS). Three studies were excluded due to the lack of a control group. Two studies [41, 42] had the same study population and reported mostly the same results as a previously included study by Rogaczewska et al. [43]; therefore, they were excluded; however, the non-repetitive data in one of these studies [10] was used for analysis. Eventually, the study by Lanzillo et al., printed in 2019, was omitted, because it was the 1-year follow-up of an already included study with the same participants and baseline characteristics [44]. The study selection process, which is depicted in Fig. 2, yielded the final inclusion of 18 studies with a total of 1552 evaluated eyes in MS cases (673 MSON eyes, 741 MSNON eyes, and 138 eyes without specification for the presence of ON) in addition to 1107 evaluated control eyes [22, 29, 31, 36, 43, 45–57]. Two studies did not report the data on the MS group as a whole, so the data on the MSON group of these studies were used in the MS vs. HC analyses [43, 56].

### Study characteristics

A complete summary of the included studies’ characteristics is illustrated in Table 1. All of the 18 studies had observational designs, five of which were conducted in the United States [22, 46, 48, 49, 57], three in Italy [31, 52, 54], three in Turkey [45, 47, 55], one in China [29], one in Korea [50], one in Poland [43], one in Germany [56], one in Portugal [51], one in Egypt [36] and one in Spain [53]. In regard to MS diagnosis criteria, eight articles used the 2010 revised McDonald Criteria [22, 31, 45, 47, 48, 53, 55, 57], eight articles used the 2017 revised McDonald Criteria [29, 36, 43, 49–51, 54, 56], a single study used the 2005 revised McDonald Criteria [46], and finally, one study did not report any specific criteria [52]. Nine studies selected their case exclusively from the RRMS subtype of patients [19, 36, 43, 45, 47, 49, 53, 54, 56]. The rest of the studies did not specify the subtype of MS cases. All included studies divided the cases into MSON and MSNON groups except for three studies [50, 54, 55]. Unlike other studies, there was no healthy control (HC) group in the study by Murphy et al., and the comparisons and analyses were made between MSON and MSNON subjects in this study [49]. Various ophthalmological and general health-related exclusion criteria for each study, as well as the method of eye selection, pupil dilation status, and the items for which cases and controls were adjusted, are depicted in Additional file 3: Table S3.

**Table 1:** Overview of included studies; demographic and subject characteristics

First author/year	MS criteria	ON criteria	Additional scoring	Trait	Number of traits	Men (%)	Age (years)	Disease duration (years)	BCVA of enrolled eyes (logMAR)	Treatment (%)	IOP of enrolled eyes	EDSS	Type of OCT-A
Aly 2022 [56]	McDonald 2017	–	EDSS, HCVA, LCVA	RRMS	21 (41 eyes)	24	38 ± 11.4	68 ± 49 months	–	90	–	1.4 ± 1.2	RTVue XR Avanti
				MSON	15 eyes								
				MSNON	26 eyes								
				HC	42 eyes	24	42 ± 9.5						
Ava 2022 [55]	McDonald 2010	–	–	MS	41 (82 eyes)	34.1	38 ± 6.4	–	0.8 ± 0.24	–	–		RTVue XR Avanti
				HC	26 (51 eyes)	42.4	36 ± 3.4		1.0 ± 0.0				
Jesus 2021 [51]	McDonald 2017	Chart documentation	EDSS	RRMS	45 (45 eyes)	68.88	41.87 ± 11.4	–	–	100	–		Heidelberg Spectralis OCTA
				MSON	19 (19 eyes)					100			
				MSNON	26 (26 eyes)					100			
				HC	45 (45 eyes)	73.33	39.36 ± 8.93						
Khader 2021 [36]	McDonald 2017	Chart documentation	–	MSON	10 eyes		31.7 ± 6.395	4.9 ± 2.846	0.978 ± 0.472	–	–		Zeiss Cirrus 4000
				MSNON	10 eyes		30 ± 5.207	3.7 ± 1.636	0.366 ± 0.133				
				HC	10 eyes		30 ± 2.981						
Lee 2021 [50]	McDonald 2017	–	–	MS	23 (36 eyes)	21.73	38	35 months	0.06 ± 0.28	–	–		Topcon DRI OCT Triton
				HC	36 (36 eyes)	36.11	42		0.00 ± 0.01				
Rogaczewska 2021 [43]	McDonald 2017	–	–	RRMS	40 (75eyes)	20	35.15 ± 7.47	8 (3–32)	0.00 (0.00–0.20)	–	–	–	RTVue XR Avanti
				MSON	30 eyes								
				MSNON	45 eyes								
				HC	20	15	37.9 ± 11.47		0.00 (0.00–0.00)				
Liu 2021 [29]	McDonald 2017	Chart documentation	–	MS	83 (146 eyes)	33	31 (27; 38)	5 (2; 9.45)	0.30 (0.07; 0.54)	72.28	–	–	Zeiss Cirrus 5000
				MSON	76 eyes								
				MSNON	70 eyes								
				HC	34	41	27 (24; 29)						
Yilmaz 2020 [45]	McDonald 2010	–	–	RRMS	47	17	38.29 ± 8.71	8.14 ± 4.19	0.02 ± 0.007	100	–	–	Nidek Advance RS-3000
				MSON	35 eyes								
				MSNON	59 eyes								
				HC	61	20	38.62 ± 11.51		0.00 ± 0.00				
Ulusoy 2020 [47]	McDonald 2010	–	MMSE; DSM-IV	RRMS	20	35	43.8 ± 12.8	9.53 ± 6.2	–	–	12.1 ± 2.4	2.29 ± 0.93	RTVue XR Avanti
				MSON	26 eyes								
				MSNON	14 eyes								
				HC	24	37.5	41.42 ± 10.81				13.5 ± 2.5		
Murphy 2020 [22]	McDonald 2010	–	MSFC; Processing speed test	MS	94 (159 eyes)	20	39.9 ± 10.6	10 (3–16)	–	100	–	1.5 (1.5–2)	Heidelberg Spectralis OCTA
				MSON	71 eyes								
				MSNON	88 eyes								
				HC	39 (71 eyes)	44	32.4 ± 11.2						
Murphy 2020 [49]	McDonald 2017	Chart documentation	–	RRMS	57				–	–	–	–	Heidelberg Spectralis OCTA
				MSON	43 (184 eyes)	16	33.7 ± 9.3	4.5 ± 6.2					
				MSNON	14 (48 eyes)	22	44.4 ± 7.7	14.9 ± 4.8					
Jiang 2020 [57]	McDonald 2010	Chart documentation	MMSE	RRMS	80 (159 eyes)	18.75	40.4 ± 10.4	8.1 ± 7	–	100	–	2.2 ± 1.8	Zeiss Cirrus 5000
				MSON	32 (36 eyes)								
				MSNON	48 (123eyes)								
				HC	99 (198 eyes)	31.31	38.6 ± 13.8						
Farci 2020 [52]	–	Chart documentation	–	MS	48 (91 eyes)	13.2	–	–	–	70	–	–	RTVue XR Avanti
				MSON	65 eyes								
				MSNON	26 eyes								
				HC	23 (29 eyes)	65.5							
Cordon 2020 [53]	McDonald 2010	–	MMSE, MSQoL-54	RRMS	92 eyes	13	41.7 ± 12.11	–	0.04 ± 0.08	–	15.37 ± 2.04	2.02 ± 1.43	Topcon DRI OCT Triton
				MSON	20 eyes								
				MSNON	72 eyes								
				HC	149 eyes	13.4	41.81 ± 18.36		0.05 ± 0.07		15.15 ± 1.57		
Cennamo 2020 [54]	McDonald 2017	–	EDSS	RRMS	10 (20 eyes)	30	29.7 ± 6.3	4 ± 1.3	–	–		2.3 ± 0.57	RTVue XR Avanti
				HC	15 (30 eyes)	33.33	28.2 ± 8.6						
Spain 2018 [48]	McDonald 2010	–	EDMUS scale	MS	45 (68 eyes)	31	45 ± 11	14 ± 10	0.04 ± 0.13	–	14 ± 2.9	–	Prototype Axsun SS-OCT
				MSON	20 (25 eyes)	25	41 ± 10	15 ± 10	0.03 ± 0.16				
				MSNON	25 (43 eyes)	36	49 ± 12	13 ± 9	0.04 ± 0.11				
				HC	32 (55 eyes)	16	41 ± 12		− 0.05 ± 0.10		15 ± 2.6		
Lanzillo 2018 [31]	McDonald 2010	Chart documentation	MSSS, EDSS	MS	50 (100 eyes)	38	40.64 ± 12.45	11.06 ± 7.19	0.10 ± 0.23	96	–	3.50 ± 1.28	RTVue XR Avanti
				MSON	23 eyes			12.54 ± 7.16	0.16 ± 0.26			3.60 ± 1.27	
				MSNON	77 eyes			10.62 ± 7.19	0.08 ± 0.21			3.46 ± 1.28	
				HC	46 (92 eyes)	48	43.33 ± 12.86		0.02 ± 0.05				
Wang 2014 [46]	McDonald 2005	Chart documentation	EDSS, EDMUS scale	MS	35 (52 eyes)	–	40 ± 8.9	17 ± 10.4	0.01 ± 0.1	–	14 ± 2.2	3.2 ± 1.9	Prototype Axsun SS-OCT
				MSON	10 (14 eyes)		47 ± 12.7	12 ± 8.4	0.04 ± 0.1		15 ± 3.1	3.8 ± 1.7	
				MSNON	25 (38 eyes)		50 ± 9.6		0.01 ± 0.1		15 ± 2.3		
				HC	21 (21 eyes)								

–: Data not reported

*EDSS* expanded disability status scale, *HCVA* high-contrast visual activity, *LCVA* high-contrast visual acuity, *BCVA* best corrected visual acuity, *MMSE* mini-mental state examination, *DSM-IV* diagnostic and statistical manual of mental disorders, fourth edition, *MSFC* multiple sclerosis functional composite, *MSQoL-54* multiple sclerosis quality of life-54, *EDMUS* European database for multiple sclerosis, *MSSS* multiple sclerosis severity score, *IOP* INTRA ocular pressure, *OCT-A* optical coherence tomography angiography, *RRMS* relapsing remitting MS, *MSON* multiple sclerosis with optic neuritis, *MSNON* multiple sclerosis without optic neuritis, *HC* healthy controls, *MS* multiple sclerosis, *ON* optic neuritis

In seven studies, OCT-A measurements were conducted using the Optovue RTVue XR Avanti (Optovue Inc., Fremont, CA) with AngioVue software [31, 43, 47, 52, 54–56]. Moreover, three studies used the Zeiss Cirrus (Carl Zeiss Meditec, Inc., Dublin, CA) with AngioPlex software [29, 36, 57], three studies used Heidelberg Spectralis OCT-A (Heidelberg Engineering, Heidelberg, Germany) with internal Heidelberg software [22, 49, 51], two studies used prototype Axsun Swept Source (SS)–OCT Engine (Excelitas Technologies Corp., Billerica, MA) with OCT Host software [46, 48], two studies used Topcon DRI OCT Triton Plus (Topcon Corp., Tokyo, Japan) with Topcon IMAGEnet software [50, 53], and one study used Nidek Advance RS-3000 (Nidek Co., Gamagori, Japan) with AngioScan software [45]. Two studies employed ImageJ software (https://imagej.nih.gov/ij/) as well as automated internal software of OCT-A device for quantitative analysis of images [22, 51].

### Vessel density in optic disc area

**Fig. 3:**
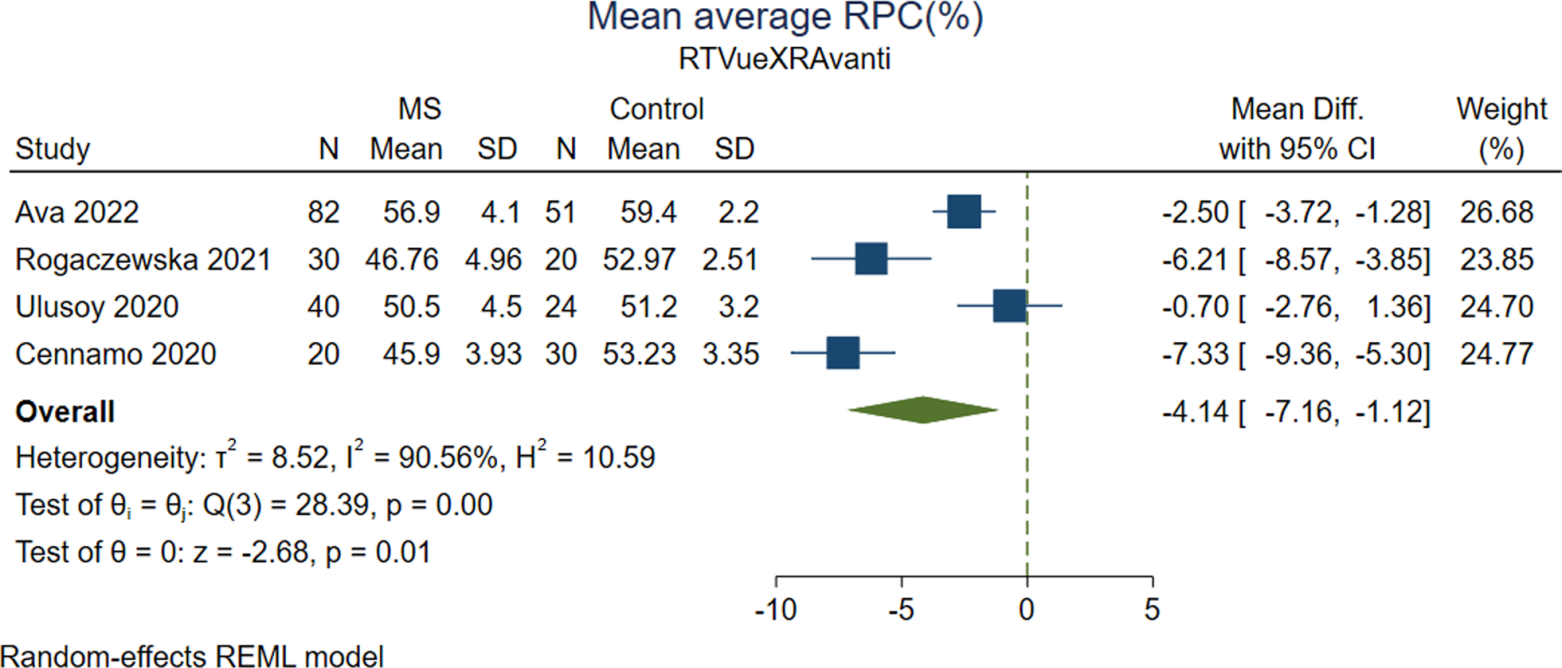
Forrest plot of the meta-analysis for the mean average RPC% between MS cases and healthy controls using Optovue machine. The meta-analysis was conducted with a random-effects model. The size of the square for each article demonstrates the attributed weight, and the horizontal line indicates the 95% confidence interval (CI). The diamonds show the standardized mean difference, and their width represents the 95% CI. N, number of subjects; SD, standard deviation; RPC: radial peripapillary capillary; MS: multiple sclerosis

Comparison of average RPC vessel density between MS vs. HC showed inconsistent results in studies with different OCT-A machines. Average RPC vessel density was reported in four Optovue studies [43, 47, 54, 55], two Topcon studies [50, 53], one Zeiss study [36], and one Nidek study [45] (Tables 2 and 3). Results of analysis of four Optovue studies measuring whole image RPC vessel density in 172 MS and 125 HC eyes indicated significantly lower values for the MS group (MD, − 4.137; 95% CI − 7.158 to − 1.115; *P* = 0.007; *I*^2^ = 90.56%) (Fig. 3). Likewise, analysis of two Optovue studies [43, 47] that evaluated whole image RPC vessel density in 56 MSON, 59 MSNON, and 88 HC eyes did not show any differences between MSON vs. HC (*P* = 0.12) and MSNON vs. HC (*P* = 0.43). However, the analysis of MSON vs. MSNON revealed significantly reduced average RPC vessel density for MSON group (MD, − 2.702; 95% CI − 4.239 to − 1.166; *P* = 0.0006; *I*^2^ = 0.00%). Regarding optic disc area quadrants, MSON eyes had significantly decreased mean RPC vessel density compared to MSNON eyes in superior (MD, − 2.313; 95% CI − 4.172 to − 0.454; *P* = 0.01; *I*^2^ = 0.00%), temporal (MD, − 4.317; 95% CI − 6.349 to − 2.287; *P* < 0.0001; *I*^2^ = 6.15%), and nasal (MD, − 3.270; 95% CI − 5.068 to − 1.471; *P* = 0.0004; *I*^2^ = 0.00%) sectors, but the difference in inferior quadrant was insignificant (*P* = 0.11).

**Table 2:** Direction of effects in cases vs. HC

Study (Optovue)	FAZ area	Superficial foveal VD	Superficial parafoveal VD	Superficial perifoveal VD	Superficial whole VD	Deep fovea VD	Deep parafoveal VD	Deep perifoveal VD	Deep whole VD	Macula	Optic disc	Choriocapillaris layer	RPC peripapillary VD	RPC whole VD
Aly et al. [56]	↑MS/MSON	–	–	–	↓MS/MSON	–	–	–	–	–	–	–	–	–
Ava et al. [55]	–	–	–	–	–	–	–	–	–	–	↓Whole image, inside disc ONH VD: MS ↓Whole image, inside disc, and peripapillary RPC VD: MS	–	–	–
Rogaczewska et al. [43]	–	–	–	–	–	–	–	–	–	–	–	–	↓MS/MSON/MSNON	-
Ulusoy et al. [47]	–	–	↓MS/MSON ∼MSNON	↓MS/MSON ∼MSNON	↓MS/MSON ∼MSNON	–	∼MS/MSON/MSNON	∼MS/MSON/MSNON	∼MS/MSON/MSNON	–	↓ Inf-Temp: MS/MSON ∼MSNON	–	–	–
Farci et al. [52]	–	–	↓MS	↓MS	↓MS	↓MS	–	–	–	–	–	↑MS	–	–
Cennamo et al. [54]	–	–	–	–	↓MS	–	–	–	∼MS	–	–	∼MS	–	↓MS
Lanzillo et al. [31]	–	↓MS/MSON/MSNON	↓MS/MSON/MSNON	–	↓MS/MSON/MSNON	–	–	–	–	–	–	–	–	–

Study (Zeiss)	FAZ area	Superficial foveal VD/PD	Superficial parafoveal VD/PD	Superficial perifoveal VD/PD	Superficial whole VD/PD	Deep fovea VD/PD	Deep parafoveal VD/PD	Deep perifoveal VD/PD	Deep whole VD/PD	Macula	Optic disc	Choriocapillaris layer	RPC peripapillary VD/PD	RPC whole VD/PD
Khader et al. [36]	–	–	–	–	↓MSON/MSNON	–	–	–	↓MSON/MSNON	–	↓VD:MSON/MSNON	–	–	–
Liu et al. [29]	∼MS	–	–	–	∼MS ↓MSON/MSNON	–	–	–	–	–	–	–	–	–
Jiang et al. [57]	–	–	–	–	↑MS/MSNON/VVD MSON ∼MSON	–	–	–	↑ VVD MSON/VVD MSNON	–	–	–	–	↑ MS/MSNON/VVD MSON/VVD MSNON ∼MSON

Study (Nidek)	FAZ area	Superficial foveal VD	Superficial parafoveal VD	Superficial perifoveal VD	Superficial whole VD	Deep fovea VD	Deep parafoveal VD	Deep perifoveal VD	Deep Whole VD	Macula	Optic disc	Choriocapillaris layer	RPC peripapillary VD	RPC whole VD
Yilmaz et al*.* [45]	∼MS	–	↓MS	↓MS	↓MS	–	↓MS	↓MS	↓MS	–	–	–	∼MS ↓ Temp: MS	–

Study (Heidelberg)	FAZ area	Superficial foveal VD	Superficial parafoveal VD	Superficial perifoveal VD	Superficial whole VD	Deep fovea VD	Deep parafoveal VD	Deep perifoveal VD	Deep whole VD	Macula	Optic disc	Choriocapillaris layer	Choroid	RPC whole VD
Jesus et al. [51]	–	–	–	–	–	–	–	–	–	–	–	∼MS/MSON/MSNON	↓0–1500 μm: MS/MSON/MSNON	–
Murphy et al. [22]	–	–	–	–	↓MS/MSON/MSNON	-	–	–	∼MS	–	–	–	–	–
Murphy et al. [49]	–	–	–	–	-	-	–	–	–	–	–	–	–	–

Study (Axsun)	FAZ area	Superficial foveal VD	Superficial parafoveal VD	Superficial perifoveal VD	Superficial whole VD	Deep fovea VD	Deep parafoveal VD	Deep perifoveal VD	Deep whole VD	Macula	Optic disc	Choriocapillaris layer	RPC peripapillary VD	RPC whole VD
Spain et al. [48]	–	–	–	–	–	–	–	–	–	–	↓ Flow Index: MS/MSON/MSNON	–	–	–
Wang et al. [46]	–	–	∼ Flow Index: MS/MSON/MSNON	–	–	–	∼ Flow Index: MS/MSON/MSNON	–	–	–	↓ Flow Index: MSON ∼ Flow Index: MSNON	–	–	–

Study (Topcon)	FAZ area	Superficial foveal VD	Superficial parafoveal VD	Superficial perifoveal VD	Superficial whole VD	Deep fovea VD	Deep parafoveal VD	Deep Perifoveal VD	Deep Whole VD	Macula	Optic Disc	Choriocapillaris layer	RPC peripapillary VD	RPC whole VD
Lee et al. [50]	–	–	–	–	↓MS	–	–	–	↓Inf: MS	–	–	–	–	↓Inf, Sup, Temp: MS
Cordon et al. [53]	–	–	–	↓MS	↓MS	–	–	–	–	↓Sup, inf, nasal: MS ↓ Sup, nasal: MSON/MSNON	–	–	–	↑ MS

–: Data not reported

∼: No significant difference

*OCT-A* optical coherence tomography angiography, *FAZ* foveal avascular zone, *RRMS* relapsing remitting MS, *MSON* multiple sclerosis with optic neuritis, *MSNON* multiple sclerosis without optic neuritis, *HC* healthy controls, *MS* multiple sclerosis, *VD* vessel area density, *RPC* radial peripapillary capillary, *ONH* optic nerve head, *PD* vessel perfusion density, *VVD* volumetric vessel density, *Inf* inferior quadrant, *Temp* temporal quadrant, *Sup* superior quadrant, *Nasal* nasal quadrant

**Table 3:** Direction of effects in MSON vs. MSNON

Study (Optovue)	FAZ area	Superficial foveal VD	Superficial parafoveal VD	Superficial perifoveal VD	Superficial whole VD	Deep fovea VD	Deep parafoveal VD	Deep perifoveal VD	Deep whole VD	Optic disc	Choriocapillaris layer	RPC peripapillary VD	RPC whole VD	
Aly et al. [56]	–	–	–	–	–	–	–	–	–	–	–	–	–	
Rogaczewska et al. [43]	–	–	–	–	–	–	–	–	–	–	–	↓	–	
Ulusoy et al. [47]	–	–	↓	↓	↓		∼	∼	∼			↓ Inf-temp	–	
Farci et al. [52]	–	–	–	–	–	–	–	–	–	–	–	–	∼	
Lanzillo et al. [31]	–	∼	∼ except in ETDRS inf	–	∼	–	–	–	–	–	–	–	–	

Study (Zeiss)	FAZ area	Superficial foveal VD/PD	Superficial parafoveal VD/PD	Superficial perifoveal VD/PD	Superficial whole VD/PD	Deep fovea VD/PD	Deep parafoveal VD/PD	Deep perifoveal VD/PD	Deep whole VD/PD	Macula	Optic disc	Choriocapillaris layer	RPC peripapillary VD/PD	RPC whole VD/PD
Khader et al. [36]	–	–	–	–	↓VD	–	–	–	↓VD	–	↓VD	–	–	–
Jiang et al. [57]	–	–	–	–	↑VVD	–	–	–	↓ ↑VVD	–	–	–	–	↓ ↑VVD

Study (Nidek)	FAZ area	Superficial foveal VD	Superficial parafoveal VD	Superficial perifoveal VD	Superficial whole VD	Deep fovea VD	Deep parafoveal VD	Deep perifoveal VD	Deep whole VD	Macula	Optic disc	Choriocapillaris layer	RPC peripapillary VD	RPC whole VD
Yilmaz et al. [45]	∼	–	↓	↓	↓	–	∼	∼	∼	–	–	–	∼	–

Study (Heidelberg)	FAZ area	Superficial foveal VD	Superficial parafoveal VD	Superficial perifoveal VD	Superficial whole VD	Deep fovea VD	Deep parafoveal VD	Deep perifoveal VD	Deep whole VD	Macula	Optic disc	Choriocapillaris layer	RPC peripapillary VD	RPC whole VD
Jesus et al. [51]	–	–	–	–	–	–	–	–	–	–	–	–	–	–
Murphy et al. [22]	–	–	–	–	↓	–	–	–	∼	–	–	–	–	–
Murphy et al. [49]	–	–	–	–	↓ IED	–	–	–	–	–	–	–	–	–

Study (Axsun)	FAZ area	Superficial foveal VD	Superficial parafoveal VD	Superficial perifoveal VD	Superficial whole VD	Deep fovea VD	Deep parafoveal VD	Deep perifoveal VD	Deep whole VD	Macula	Optic disc	Choriocapillaris layer	RPC peripapillary VD	RPC whole VD
Spain et al. [48]	–	–	–	–	–	–	–	–	–	–	↓ Flow Index	–	–	–
Wang et al. [46]	–	–	∼Flow Index	–	–	–	∼Flow Index	–	–	–	↓ Flow Index	–	–	–

Study (Topcon)	FAZ area	Superficial foveal VD	Superficial parafoveal VD	Superficial perifoveal VD	Superficial whole VD	Deep fovea VD	Deep parafoveal VD	Deep perifoveal VD	Deep whole VD	Macula	Optic disc	Choriocapillaris layer	RPC peripapillary VD	RPC whole VD
Cordon et al. [53]	–	–	–	–	–	–	–	–	–	–	–	–	–	–

–: Data not reported

∼: No significant difference

*MSON* multiple sclerosis with optic neuritis, *MSNON* multiple sclerosis without optic neuritis, *VD* vessel area density, *VVD* volumetric vessel density, *RPC* radial peripapillary capillary, *PD* vessel perfusion density, *VVD* volumetric vessel density, *Inf* inferior quadrant, *Temp* temporal quadrant, *Sup* superior quadrant, *Nasal* nasal quadrant

Cordon et al. [53] with the Topcon machine revealed no significant difference in average vessel density of optic disc area in MSON or MSNON vs. HC, as well as MSON vs. MSNON analyses (all *P* > 0.05). Analysis of two Topcon studies reporting mean RPC vessel densities in superior, inferior, temporal, and nasal quadrants of 128 MS and 185 HC eyes showed no significant differences in the quadrants between these groups (all *P* > 0.05). However, it should be noted that one of these two studies [50] that had a smaller field of view (4.5 × 4.5 mm^2^) reported significantly decreased mean RPC vessel density for MS eyes compared to HC in all quadrants except for the nasal quadrant (all *P* < 0.05).

The study by Yilmaz et al. [45] with the Nidek machine found no significant difference among the study groups for average RPC vessel density. The single significant difference was for the temporal quadrant, which was lower in the MS group compared to HC (*P* < 0.001).

A Zeiss study conducted by Khader et al. [36] also showed that average, superficial, and deep vascular density in the optic disc area were significantly lower in MSON and MSNON compared to the HC control group (all* P* < 0.001).

### Vessel density in the macular SCP

**Fig. 4:**
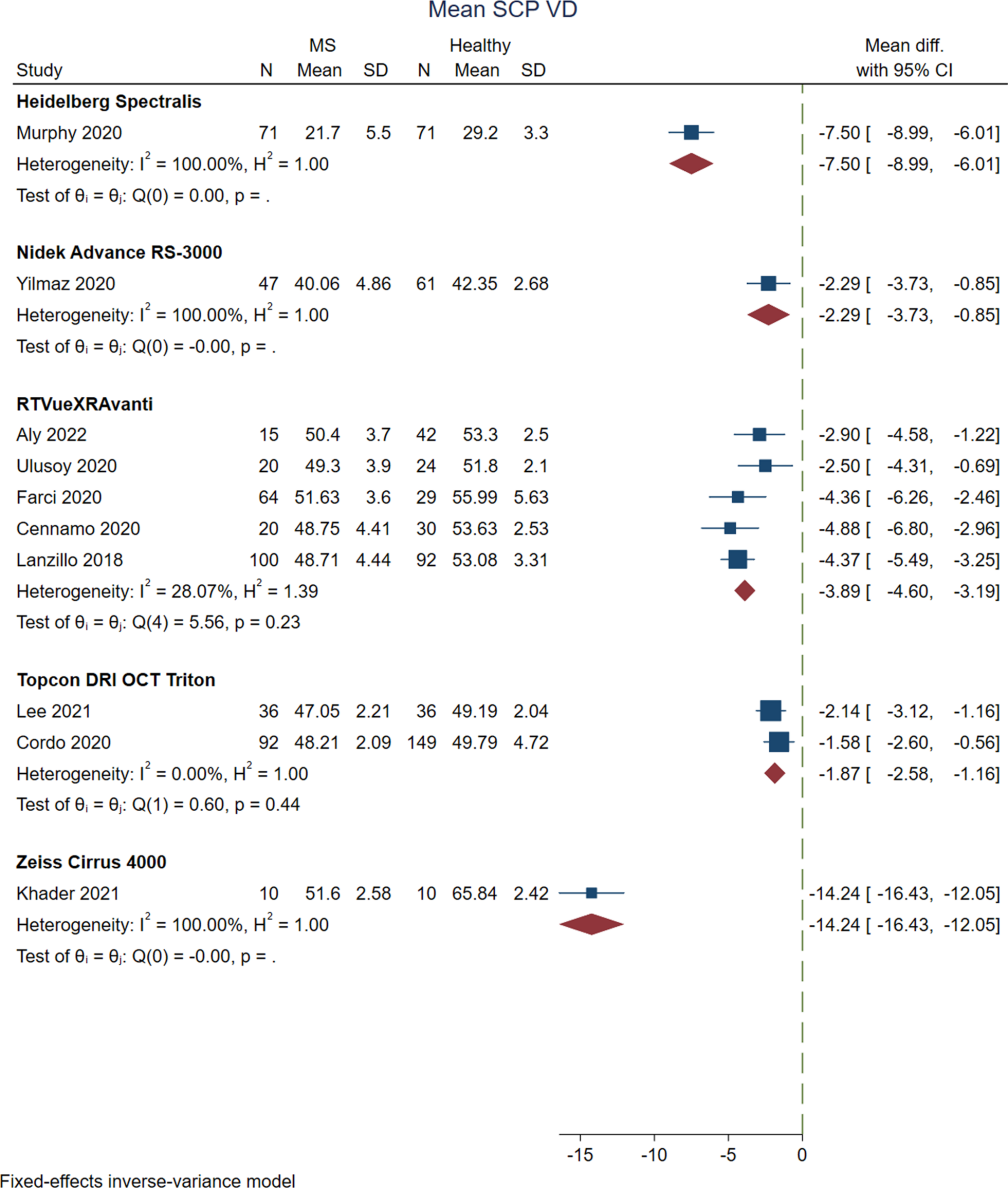
Forrest plot of the meta-analysis for the mean SCP vascular density between MS cases and healthy controls. The meta-analysis was conducted with a fixed-effects model. The size of the square for each article demonstrates the attributed weight, and the horizontal line indicates the 95% confidence interval (CI). The diamonds show the standardized mean difference, and their width represents the 95% CI. N, number of subjects; SD, standard deviation; SCP: superficial capillary plexus; MS: multiple sclerosis

Measurements of density in SCP were reported in different macular areas, mostly indicating lower values for MS cases compared to HC (Tables 2 and 3). Vessel density in the superficial layer of the macula was reported by five Optovue studies [31, 47, 52, 54, 56], three Zeiss studies [29, 36, 57], a Nidek study [45], two Heidelberg studies [22, 49], and two Topcon studies [50, 53]. Analysis of this variable between MS vs. HC by pooling the results of Optovue studies showed that MS cases had significantly decreased macular whole image vessel density in the superficial layer (MD, − 3.895; 95% CI − 4.600 to − 3.189; *P* < 0.0001; *I*^2^ = 28.07%) (Fig. 4). MS cases also had significantly lower values of SCP vessel density than the HC group in the parafoveal region (MD, − 2.637; 95% CI − 5.184 to − 0.040; *P* = 0.04; *I*^2^ = 84.93%), superior hemisphere (MD, − 2.995; 95% CI − 4.503 to − 1.486; *P* = 0.0001; *I*^2^ = 62.56%), and inferior hemisphere (MD, − 3.221; 95% CI − 4.847 to − 1.594; *P* = 0.0001; *I*^2^ = 67.11%). In a similar trend, MSON cases showed significantly less macular SCP vessel density than the HC group in the whole image (MD, − 3.762; 95% CI − 4.632 to − 2.891; *P* < 0.0001; *I*^2^ = 18.06%), parafoveal (MD, − 3.722; 95% CI − 4.851 to − 2.594; *P* < 0.0001; *I*^2^ = 0.00%), superior hemisphere (MD, − 3.823; 95% CI − 4.872 to − 2.774; *P* < 0.0001; *I*^2^ = 0.00%), inferior hemisphere (MD, − 3.909; 95% CI − 5.032 to − 2.787; *P* < 0.0001; *I*^2^ = 0.00%), superior quadrant (MD, − 5.037; 95% CI − 6.434 to − 3.640; *P* < 0.0001; *I*^2^ = 0.00%), and inferior quadrant (MD, − 3.923; 95% CI − 5.300 to − 2.547; *P* < 0.0001; *I*^2^ = 11.86%) analyses. In MSNON vs. HC analyses, the results revealed that MSNON had less macular SCP vessel density in the whole image (MD, − 2.668; 95% CI − 4.594 to − 0.743; *P* = 0.01; *I*^2^ = 82.76%), parafovea (MD, − 2.835; 95% CI − 5.318 to − 0.352; *P* = 0.03; *I*^2^ = 77.17%), superior (MD, − 2.948; 95% CI − 5.320 to − 0.576; *P* < 0.0001; *I*^2^ = 0.00%) and inferior hemispheres (MD, − 3.073; 95% CI − 5.503 to − 0.643; *P* = 0.01; *I*^2^ = 78.55%), as well as superior (MD, − 4.554; 95% CI − 5.882 to − 3.226; *P* < 0.0001; *I*^2^ = 0.00%) and temporal quadrants (MD, − 3.550; 95% CI − 4.809 to − 2.291; *P* < 0.0001; *I*^2^ = 25.65%). In the comparison of MSON vs. MSNON, none of the differences comprising the whole image, parafovea, superior and inferior hemispheres, and superior and temporal quadrants were statistically significant (all *P* > 0.05). There was no significant difference in foveal SCP vessel density between the analyzed groups (all *P* > 0.05).

The analysis of two Topcon studies [50, 53] with 128 MS and 185 HC eyes indicated that patients with MS had significantly lower macular SCP vessel densities on average (MD, − 1.871; 95% CI − 2.579 to − 1.163; *P* < 0.0001; *I*^2^ = 0.00%) (Fig. 4) as well as all of the quadrants: superior (MD, − 2.281; 95% CI − 3.198 to − 1.364; *P* < 0.0001; *I*^2^ = 0.00%), inferior (MD, − 2.053; 95% CI − 3.028 to − 1.079; *P* < 0.0001; *I*^2^ = 0.00%), temporal (MD, − 1.133; 95% CI − 1.871 to − 0.394; *P* < 0.0001; *I*^2^ = 0.00%), nasal (MD, − 1.062; 95% CI − 2.722 to − 1.082; *P* < 0.0001; *I*^2^ = 0.00%). Moreover, Cordon et al. [53], with the Topcon machine, found a significant decrease in the whole image, nasal, and superior perifoveal SCP vessel density for MSON and MSNON eyes when compared to controls (all *P* < 0.05).

Comparing MS eyes with HC (Table 2), Yilmaz et al. [45] with the Nidek machine showed lower whole image, perifoveal, and parafoveal SCP vessel densities (all *P* < 0.05). Among the studies with the Zeiss machine, while Liu et al. [29] stated no significant difference between these groups for vessel density (*P* = 0.07) and perfusion density (*P* = 0.17), Jiang et al. [57] showed that MS eyes had significantly higher vessel density (Dbox) and volumetric vessel density (Dbox/mm^3^) compared to healthy eyes (all *P* < 0.05).

In the comparison of MSON or MSNON groups with HC (Table 2), Murphy et al. [22] with the Heidelberg machine and Liu et al. [29] with the Zeiss machine showed significantly reduced whole image macular SCP vessel densities in MSON and MSNON eyes (all *P* < 0.05). In the study by Jiang et al. [57] with the Zeiss machine, only the MSNON group had significantly higher vessel density (Dbox) compared to HC; on the other hand, only the MSON group showed a significant increase in volumetric vessel density (Dbox/mm^3^) in comparison with HC (all *P* < 0.05).

Comparing the MSON group with the MSNON group (Table 3), Murphy et al. [22] with the Heidelberg machine showed significantly lower whole image macular SCP vessel density in the MSON group (*P* < 0.001). The other Heidelberg study [49] in this review indicated lower whole image macular SCP vessel density inter-eye difference (using the ON eye as the reference eye in MSON patients and the right eye as the reference eye in MSNON patients) in the MSON group (*P* = 0.002). Yilmaz et al. [45], with the Nidek machine, found significantly lower values for the whole image, perifoveal, and parafoveal SCP vessel densities in MSON patients (all *P* < 0.05). The study by Jiang et al. [57], with the Zeiss machine, showed that the volumetric vessel density of macular SCP was significantly higher in MSON cases (*P* = 0.001).

### Vessel density in the macular DCP

Analysis of vessel density in the macular DCP between MS cases and HC mainly showed insignificant differences. Vessel density measurements in the macular DCP were reported in four Optuvue [47, 52, 54, 56], two Zeiss [36, 57], one Nidek [45], one Topcon [50], and one Heidelberg [22] study (Tables 2 and ). Pooling the results of three Optuvue studies [47, 54, 56] with 75 MS and 96 HC eyes revealed no significant difference in mean macular DCP vessel density between these groups (*P* = 0.002). Analyses on the Optovue studies showed that MSON had significantly lower foveal DCP vessel density (MD, − 6.095; 95% CI − 8.739 to − 3.450; *P* < 0.0001; *I*^2^ = 85.16%) and higher parafoveal DCP vessel density (MD, 2.121; 95% CI 0.257 to 3.986; *P* = 0.03; *I*^2^ = 0.00%) compared to the HC group. Moreover, MSNON cases had no significant difference with the HC group in the whole image, foveal, and parafoveal DCP vessel densities (all *P* > 0.05). Comparing MSON with MSNON cases, the former only had significantly higher whole image macular DCP vessel density (MD, 0.856; 95% CI 0.004 to 1.709; *P* = 0.049; *I*^2^ = 0.00%), and there was no significant difference in the fovea, parafovea, and the quadrants (all *P* > 0.05).

Yilmaz et al. [45], using the Nidek machine, showed significantly lower values in MS eyes (MSON + MSNON) for the whole image, perifoveal and parafoveal DCP vessel densities compared to controls (all *P* < 0.001). However, MSON eyes did not differ significantly from MSNON eyes. Furthermore, the study by Jiang et al. [57] using the Zeiss machine and monofractal analysis (Dbox) found significantly increased volumetric vessel density of macular DCP in MSON and MSNON eyes compared to controls (all *P* < 0.05). Moreover, while vessel density (Dbox) was significantly lower in the MSON group than in the MSNON group (*P* = 0.02), volumetric vessel density (Dbox/mm^3^) was lower in the MSNON group (*P* = 0.001).

### Vessel density in the choriocapillaris

Studies with different machines reported inconsistent results on the comparison of choriocapillaris vessel density between MS cases and HC. Two studies using the Optovue machine [52, 54] and one study using the Heidelberg machine [51] measured choriocapillaris vessel density (Tables 2 and 3). In the study by Cennamo et al. [54], the choriocapillaris vessel density of RRMS cases was not significantly different from HC (*P* = 0.88). Nonetheless, Farci et al. [52] showed that the HC group had a significantly lower whole image and foveal choriocapillaris vessel density than the MSON group, MSNON group, and all MS cases (all *P* = 0.01). However, the difference between MSON and MSNON groups was statistically insignificant. Jesus et al. [51] showed that the choriocapillaris vessel density of MS cases at different distances from the fovea was not significantly different from the HC group (all *P* > 0.05). Furthermore, choroidal layer vessel density was significantly less in the MS group than in the HC group at 500–1000 µm of the fovea (*P* = 0.01) and 1000–1500 µm of the fovea (*P* = 0.01). In comparison between MSON vs. MSNON, the MSON group had a significantly lower choroidal layer vessel density at 0–500 µm of the fovea (*P* = 0.04).

### FAZ area

FAZ area in the included studies was mostly reported to be not significantly different in MS cases from HC. FAZ area has been reported in three studies with different devices, and a total of 281 examined MS eyes (150 MSON and 131 MSNON) and 232 HC eyes [29, 45, 56]. One of these studies reported two other FAZ metrics, including perimeter and circularity index as well [45]. The results of the studies by Liu et al. [29], which used the Zeiss machine, and Yilmaz et al. [45], which used the Nidek machine, indicated no significant difference in FAZ area between MS cases and HC (*P* = 0.07 and *P* = 0.76, respectively; Table 2). Yilmaz et al. showed that there was no significant difference between MSON vs. MSNON eyes in the FAZ area (*P* = 0.62; Table 3), as well; this study also revealed that the FAZ perimeter and circularity index of MS eyes were not significantly different from the HC group (*P* = 0.67 and *P* = 0.71, respectively). Moreover, eyes with and without ON were not significantly different for FAZ perimeter (*P* = 0.27) and circularity index (*P* = 0.11). However, the study by Aly et al. that used the Optovue machine showed significantly increased FAZ area in RRMS cases with ON compared to HC (*P* < 0.05). Aly et al. also reported that the FAZ area had a negative correlation with foveal thickness measures (*β* = − 0.003; *P* < 0.0001).

### Blood flow index

Only two studies with 196 measured eyes (39 MSON, 81 MSNON, and 76 HC) that used prototype Axsun machine had measured blood flow index [46, 48] (Tables 2 and 3). Whereas Spain et al. [48] reported the flow index of the optic nerve head (ONH), Wang et al. [46] evaluated the flow index in parafoveal SCP and DCP in addition to ONH. Results of the analysis of these two studies revealed that MSON and MSNON eyes both had slightly lower ONH flow index compared to HC with mean differences of − 0.023 (95% CI − 0.029 to − 0.017; *P* < 0.0001; *I*^2^ = 0.00%) and − 0.007 (95% CI − 0.012 to − 0.002; *P* < 0.0001; *I*^2^ = 0.00%), respectively. On an individual study basis, Wang et al. [46] reported a significant decrease in ONH flow index only for MSON eyes, unlike the other study. The study by Wang et al. [46] also demonstrated insignificant differences for parafoveal SCP and DCP flow indices between MSON or MSNON eyes with HC (all *P* > 0.05). In the comparison between MSON vs. MSNON eyes, the former had a significantly lower ONH flow index (MD, − 0.016; 95% CI − 0.023 to − 0.009; *P* = 0.0013; *I*^2^ = 0.00%). Nevertheless, Wang et al. [46] showed insignificant differences between MSON and MSNON eyes in parafoveal SCP and DCP flow indices (all *P* > 0.05) (Table 3).

## Discussion

Vascular abnormalities in the CNS have been studied in people with MS to elucidate their possible role in the development and prognosis of the disease. Hence, OCT-A has been recently used to investigate one of the most easily accessible internal body structures, i.e., the retina, which is known to be affected by MS, and quantify the vessel density and blood flow alterations in the macular and optic disc regions [58]. In the current systematic literature review, eighteen studies that included OCT-A measurements in people with MS were reviewed, and the reported data were analyzed quantitatively where possible. The most commonly reported result across the included studies was the significant decrease in macular SCP vessel density in MS eyes. The meta-analyses of studies using the same machines and software also showed that MS cases had significantly reduced whole image vessel density in the superficial layer of the macula. Likewise, MSON and MSNON eyes were also revealed to have lower macular SCP vessel densities in the whole image measurement and most of the subsectors. Macular DCP and RPC vessel densities, as well as ONH blood flow index, were also shown to have significant differences between MS cases and HC eyes.

In general, MS has been connected to vascular dysfunctions in three aspects: the higher risk of ischemic stroke in this disease, global cerebral hypoperfusion, and reduced venous vasculature [59]. However, in the retina, there may be different reasons for the observed reductions in vessel density. The SCP is defined as the vascular network (venules, capillaries, arterioles) supplying the RNFL and GCIPL [60]. It is well-documented that these layers of the retina tend to be thinned in MS eyes, even more so if there is a prior history of ON, as compared to HC [10, 61]. In addition, histopathological studies of MS eyes have demonstrated inflammation and cell atrophy in the retina and optic nerve [62]. Therefore, the decrease of vessel density in the superficial layer may simply be due to a reduction of demand for oxygen and metabolites secondary to neuroaxonal degeneration and atrophy of pRFNL and GCIPL. To support this theory, Murphy et al. showed that macular SCP vessel density has a strong correlation with GCIPL thickness, especially in MS eyes, compared to HC, which got even more prominent in MSON eyes [22]. In this regard, although four of included studies [22, 45, 47, 49] reported significantly lower macular SCP vessel density in MSON eyes compared to MSNON eyes, results of the conducted meta-analysis indicated insignificant differences. Another hypothesis suggests that the reduction of macular SCP vessel density in MS eyes is the direct result of endothelial dysfunction caused by MS or ON-induced inflammation [22].

On the other hand, it has been suggested that alterations in vessel densities may not be simply related to bystander damage caused by MS but an essential part of the pathophysiology of the disease [63]; this hypothesis may be further supported by the results of neuroimaging studies that have demonstrated hypoperfusion in some areas of the gray matter even when atrophy is absent [64]. Moreover, several investigations have stated that hypoperfusion signs in the retina had the same characteristics with reduced blood flow detected in areas with cerebral MS lesions [65–67]. Previously, Wang et al. reported that the ONH flow index was attenuated in MSON eyes compared to HC. However, no significant alterations were found in the parafoveal SCP and DCP flow indices [46]. We pooled the data of the study by Wang et al. with the findings of a more recent study [48] using a similar machine, which had reported a significantly reduced ONH flow index for MS and MSNON eyes. Results of the analysis indicated that both MSON and MSNON cases had decreased ONH flow index in the optic disc area in comparison with HC. Furthermore, MSON patients had an even more significantly attenuated ONH flow index than MSNON patients.

Spain et al. showed that the ONH flow index did not have a significant correlation with the presence of vascular risk factors suggesting that reduced perfusion may be occurring as part of MS pathophysiology rather than as a sequela of systemic disease [48]. Nevertheless, some researchers believe that reductions in vessel density occur secondary to atrophy of nerve tissue (for instance, RNFL loss) and do not precede it. Thus, vascular investigations in this manner are not more useful than structural analysis [26].

Alterations reported for vessel density of the macular DCP, which supplies the inner nuclear and outer plexiform layers, were not consistent between the included studies. Most of the studies reported insignificant differences in macular DCP vessel density for the whole image or sectors. This was confirmed by the analysis conducted in our study to compare whole image macular DCP vessel density between MS eyes and HC. However, this metric was significantly lower in MSON compared to HC and higher compared to MSNON cases. Regarding the optic disc area measurements, the results of analysis on vessel density of RPC, which is a unique vascular plexus in the RNFL [50], indicate a significant difference for MS eyes compared to HC and MSON compared to MSNON.

A study measuring hemoglobin levels in the ONH of MS patients reported that the hemoglobin percentage was decreased in MS cases in comparison with controls, especially in the temporal quadrant, which is in line with the results of the included studies in this review [68].

The study by Jiang et al. introduced and measured a novel metric called volumetric vessel density that evaluates structural and vascular alteration in the retina in a hybrid manner [57]; this study showed that MSON eyes had significantly lower macular SCP and DCP volumetric vessel density than MSNON eyes. However, volumetric vessel density is largely affected by structural changes rather than density alterations, and one might argue that separate measurements of these two aspects might be more informative and reliable [8].

There were several limitations for our systemic review. First, there is a concept that OCT-A in its current form is rather binary and only identifies the absolute absence of RBC flow in the capillaries; hence a gradual decrease of flow is not detectable by OCT-A [26]. Moreover, evidence suggests that this relatively new imaging technology suffers from imaging artifacts as the smallest ocular movements can influence measurements abundantly [69].

The other limitation of OCT-A studies is the various boundaries and segmentation algorithms used by different imaging machines and analyzing software. This vast heterogeneity made the comparison between studies and pooling the data together for meta-analysis nearly impossible. Therefore, the conducted analyses were among only two or three studies with the same machines and software.

Developing equations that could convert the findings between various analytic approaches may pave the way for a more comprehensive analysis. Moreover, all of the included studies had cross-sectional designs, while longitudinal studies may better elucidate the pathophysiology behind the alterations of vessel density in MS patients. Demographical differences such as participants’ age, disease duration, disease-modifying treatments, and methodological variance among studies, such as OCT-A timing [70, 71] or field of view, may act as sources of bias and explain the dissimilarity of findings.

## Conclusion

OCT-A provides a quantitative tool to explore alterations in the retinal and optic nerve vascular networks that might be disrupted by optic neuritis and MS. This systematic review and meta-analysis of the studies reporting OCT-A measurements of patients with MS confirmed the tendency of MS eyes to exhibit reduced vessel density in the macular and optic disc areas. As the current OCT-A techniques produce two-dimensional pictures, they are unable to truly differentiate between constriction, shrinkage, or loss of vasculature. Future technological advancements should address this problem. Further studies with larger populations, longitudinal designs, and standardized segmentation and imaging analysis protocols are required to better understand the temporality and chronology of vascular alterations occurring in MS eyes. Such advances will make the application of OCT-A more practical while potentially offering a better understanding of the pathogenesis of MS.

## Supplementary Information

**Additional file 1: Table S1.** OCT-A metrics and regions assessed in the included studies with term definitions.

**Additional file 2: Table S2.** Summary of fields of view and dimensions used to define retinal regions in included studies.

**Additional file 3: Table S3.** Exclusion criteria and additional information of included studies.

## Data Availability

The data sets used and/or analyzed during the current study are available from the corresponding author on reasonable request.
