# Supplementary material for: Optical coherence tomography angiography measurements in multiple sclerosis: a systematic review and meta-analysis
Source: J Neuroinflammation. 2023 Mar 27;20:85. doi: 10.1186/s12974-023-02763-4 (PMC10041805; doi:10.1186/s12974-023-02763-4)
Supplement: Supplementary file 1 — Additional file 1: Table S1. OCT-A metrics and regions assessed in the included studies with term definitions. [file 12974_2023_2763_MOESM1_ESM.docx]

| First author/Year | Metrics described (acronyms used) | Calculation method | Retinal layers measured (acronyms used) | Regions measured | Term | Study Definitions |
| --- | --- | --- | --- | --- | --- | --- |
| Aly 2022* [56] | Vessel Density/Foveal Avascular Zone | Angiovue | Fovea: SVC and DVC | Fovea: Segmentation of the SVC and DVC within a circle around the fovea between 1- and 3-mm eccentricity and assessment of the FAZ was performed automatically. | - | - |
| Ava 2022* [55] | Vessel Density | Angiovue | Optic Disc: ONH and RPC VD | Optic Disc: Whole image, inside optic disc and peripapillary of ONH and RPC VD | Vessel density | The area covered by the blood vessels in the region scanned using OCTA |
| Jesus 2021***** [51] | Vessel Density | Spectralis/imagej | Fovea: Vascular density of choroid and choricapillaies layer | Fovea: Vascular density of choroid and choricapillaies layer in 0-500, 500-1000, 1000-1500 to fovea regions | Vessel density | The percentage area occupied by the large vessels  And microvasculature |
| Khader 2021** [36] | Vascular Density Index | Angioplex | Optic Disc: Average, sup papillary level, deep papillary lever, outer retinal level, and choroidal level vascular density index | Optic Disc: Average, sup papillary level, deep papillary lever, outer retinal level, and choroidal level vascular density index | Sup papillary level, deep papillary lever, outer retinal level, and choroidal level | The superficial papillary level was determined to extend from a point at the level of the internal limiting membrane to a point at the outer boundary of the inner plexiform layer.  The deep papillary level was determined to extend from a point at the level of the outer boundary of the inner plexiform layer to a point at the outer edge of the outer plexiform layer.  The Outer retina level extends from a point at the level of the outer boundary of the outer plexiform layer to an end at the level of Bruch’s membrane.  Choroidal level extends from the level of Bruch’s membrane to 353 microns below it. |
| Lee 2021****** [50] | Vessel Density | Topcon imagenet | Optic disc: Superfcial retinal capillary plexus/ Deep retinal capillary plexus/ Radial peripapillary capillary | Optic disc: SRCP, DRCP and RPC (nasal, inferior, temporal, superior, whole) | Superficial retinal capillary plexus (SRCP), deep retinal capillary plexus(DRCP) | The superficial retinal capillary plexus extended from 3 µm below the internal limiting membrane (ILM) to 15 µm below the IPL  Deep retinal capillary plexus extended from 15 to 70 µm below the IPL.  The radial peripaillary capillary segment extended from the ILM to the posterior boundary of the RNFL. |
| Rogaczewska 2021* [43] | Vessel Density/Flow Density | Angiovue automated | Optic disc: radial peripapillary capillary plexus | Optic disc: Peripapillary, superior, inferior,temporal | Radial peripapillary capillary | A 1.0 mm wide round  Annulus extending outward from the optic disc boundary. |
| Liu 2021** [29] | 1) Foveal Avascular Zone  2) Vascular density  3)perfusion density | Angioplex automated | Macula: superficial capillary plexus | Macula: whole | Vessel density, perfusion density | Vessel density was defined as the total length of the perfused vasculature per unit area in a region of measurement  Perfusion density was defined as the total area of perfused vasculature per unit area in the region of measurement |
| Yilmaz 2020**** [45] | 1) Foveal Avascular Zone 2)Vascular density | Angioscan automated | Macula: superficial capillary plexus, deep capillary plexus/Optic disc: retinal peripapillary capillary plexus | Macula: whole,parafovea,perifovea / optic disc: peripapillary | Vascular Density | The percentage of the vascularized tissue within the surrounding area. |
| Ulusoy 2020* [47] | Capillary Vessel Density/ areas of capillary non perfusion/ | Angiovue automated | Macula: superficial capillary plexus/deep capillary plexus Optic Disc: peripapillary capillary vascular density | Macula: Whole, Foveal, Superior Hemisphere, Inferior Hemispher, Parafovea, Perifovea  Optic Disc: Whole, Peripapillary, Superior Hemisphere, Inferior Hemisphere, Temporal, Nasal | - | - |
| Murphy 2020***** [22] | Vessel Density | Angiovue with imagej | Macula: superficial capillary plexus/deep capillary plexus | Macula: Whole | - | - |
| Murphy 2020***** [49] | Vessel Density | - | Macula: superficial capillary plexus/deep capillary plexus | Macula: Whole | - | - |
| Jiang 2020** [57] | Vascular Plexus, vascular density, volumetric vessel density | Angioplex automated | Optic disc: retinal vascular network, volumetric vessel density of retina/macula: superficial vascular plexus,deep vascular plexus,volumetric vessel density of superficial retina, volumetric vessel density of deep retina | Macula: whole/optic disc: whole | Vascular Plexus/vascular density/ volumetric vessel density(VVD) | The vvdr was the VD of the RVN divided by the tissue volume (the same circular area) from the RNFL to the OPL.  VD measurements of the SVC and DVC divided by the corresponding tissue volumes (with a diameter of 2.5 mm). |
| Farci 2020* [52] | Flow density/capillary plexus | Angioanalytics automated | Macula: superficial capillary plexus/deep capillary plexus/retinal capillary flow density--choriocapillaries layer-- | Macula:Whole, Foveal, Superior Hemisphere, Inferior Hemisphere ,Parafovea, Perifovea, temporal, nasal | Retinal capillary flow density(CFD) | As the percentage of the sample area occupied by vessel lumens after intensity thresholding and segmentation of images. |
| Cordon 2020****** [53] | Vascular Density | Topcon imagenet | Macula/Optic disc | Macula: sup, inf, nasal, temp, central/Optic disc: central, nasal, inf, sup, temp | Vessel density | The surface, measured in mm2, which limits  The cube in which erythrocyte movement is detected. |
| Cennamo 2020* [54] | Vascular density | Angiovue automated | Macula: superficial capillary plexus/deep capillary plexus-Optic disc: radial peripapillary capillary plexus | Macula: Whole/Optic Disc: Whole | VD for RPC | Was analyzed in the superficial retinal layers and extended from the inner layer membrane to the retinal nerve fiber layer posterior boundary. |
| Spain 2018*** [48] | Flow Index | SS OCT Angio automated | Optic Disc | Optic disc: whole | Flow index | Corresponded to the area (or calibre) of large vessels and both the area (or vessel density) and velocity of capillaries. |
| Lanzillo 2018* [31] | Vessel Density | Angiovue automated | Superficial Capillary Layer | Macula: fovea, parafovea | Superficial Capillary layer, vessel density | Superficial capillary layer was defined by starting with the internal limiting  Membrane and by selecting a thickness sufficient to  Include the ganglion cell layer in the central macular  Region.  Vessel density was defined as the percentage  Area occupied by the large vessels and microvasculature in the analysed region. |
| Wang 2014*** [46] | Flow Index | SS OCT Angio automated | Optic disc, parafovea of retina | Optic disc: whole, parafovea of retina | ONH flow index, parafovea retinal flow index | ONH FI was defined as the average flow signal (decorrelation value) within the whole ONH.  Parafoveal retinal FI was defined as the average flow signal within the annular zone of 0.6 to 2.6 mm diameter around the foveal centre. |

* indicates studies using an Optovue machine, ** indicates studies using a Zeiss machine. *** indicates studies using a prototype Axsun SS-OCT machine, **** indicates studies using a Nidek machine, ***** indicates studies using a Heidelberg machine, and ****** indicates studies using a Topcon machine

–: Data not reported.

Acronyms used in the table: OCT-A: Optical coherence tomography angiography/ RRMS: Relapsing remitting MS/ MSON: Multiple sclerosis with Optic neuritis/ MSNON: Multiple sclerosis without Optic neuritis/ HC: Healthy controls/MS: Multiple sclerosis/ VD: Vessel area density / VVD: Volumetric vessel density/RPC: Radial peripaillary capillary/ PD: Vessel perfusion density/ VVDr: VVD of retina/ IED: Inter eye difference/ Inf: Inferior quadrant/ Temp: Temporal quadrant/ Sup: Superior quadrant/ Nasal: Nasal quadrant/ MOGAD: MOG-antibody associated disease / BCVA: Best corrected visual acuity / ONH: Optic nerve head/ MRI: Magnetic resonance imaging/ ON: Optic neuritis/ IOP: Intra ocular pressure/ EDSS: Expanded Disability Status Scale score / EMDUS: physician-rated European Database for Multiple Sclerosis grading score/ SVC: Superficial vascular complex/ DVC: Deep vascular complex/ FAZ: Foveal avascular zone/ SRCP: Superficial retinal capillary plexus/ DRCP: Superficial retinal capillary plexus/ RNFL: Retinal nerve fiber layer/ FI: Flow index/ VVDd: VVD of deep vascular complex/ VVDs: VVD of superficial vascular complex
