# Supplementary material for: Optical coherence tomography angiography measurements in multiple sclerosis: a systematic review and meta-analysis
Source: J Neuroinflammation. 2023 Mar 27;20:85. doi: 10.1186/s12974-023-02763-4 (PMC10041805; doi:10.1186/s12974-023-02763-4)
Supplement: Supplementary file 2 — Additional file 2: Table S2. Summary of fields of view and dimensions used to define retinal regions in included studies. [file 12974_2023_2763_MOESM2_ESM.docx]

|  |  | Macula | | | | | | | |  | Optic disc | | |
| --- | --- | --- | --- | --- | --- | --- | --- | --- | --- | --- | --- | --- | --- |
|  |  | Superficial | | | | Deep | | | |  | Superficial | Radial peripapillary capillary | |
| **First author/Year** | **Field(s) of view** | Whole | Fovea | Parafovea | Perifovea | Whole | Fovea | Parafovea | Perifovea | **Field(s) of view** | Peripapillary | Whole | Peripapillary |
| Aly 2022* [56] | **-** | **-** | 6 x 6 mm2 | **-** | **-** | **-** | 6 x 6 mm2 | **-** | **-** | **-** | **-** | **-** | **-** |
| Ava 2022* [55] | **-** | **-** | **-** | **-** | **-** | **-** | **-** | **-** | **-** | 3 x 3 mm2 | **-** | **-** | **-** |
| Jesus 2021***** [51] | **-** | **-** | **-** | **-** | **-** | **-** | **-** | **-** | **-** | **-** | **-** | **-** | **-** |
| Khader 2021** [36] | **-** | **-** | **-** | **-** | **-** | **-** | **-** | **-** | **-** | **-** | **-** | **-** | **-** |
| Lee 2021****** [50] | 3 x 3 mm2 | **-** | **-** | **-** | **-** | **-** | **-** | **-** | **-** | 3 x 3 mm2 | **-** | **-** | **-** |
| Rogaczewska 2021* [43] | **-** | **-** | **-** | **-** | **-** | **-** | **-** | **-** | **-** | 4.5 x 4.5mm2 | **-** | **-** | 4.5 x 4.5mm2 |
| Liu 2021** [29] | 6 x 6 mm2 | 6 x 6 mm2 | **-** | **-** | **-** | **-** | **-** | **-** | **-** | **-** | **-** | **-** | **-** |
| Yilmaz 2020**** [45] | 3 x 3mm2 | 3 x 3mm2 | **-** | 3 x 3mm2 | 3 x 3mm2 | 3 x 3mm2 | **-** | 3 x 3mm2 | 3 x 3mm2 | 2.4 x 4 mm2 | **-** | 2.4 x 4 mm2 | **-** |
| Ulusoy 2020* [47] | 4.5 x 4.5 mm2 | 4.5 x 4.5 mm2 | 4.5 x 4.5 mm2 | 4.5 x 4.5 mm2 | 4.5 x 4.5 mm2 | 4.5 x 4.5 mm2 | 4.5 x 4.5 mm2 | 4.5 x 4.5 mm2 | 4.5 x 4.5 mm2 | 4.5 x 4.5 mm2 | 4.5 x 4.5 mm2 | **-** | **-** |
| Murphy 2020***** [22] | **-** | **-** | **-** | **-** | **-** | **-** | **-** | **-** | **-** | **-** | **-** | **-** | **-** |
| Murphy 2020***** [49] | 3 x 3mm2 | 3 x 3mm2 | **-** | **-** | **-** | **-** | **-** | **-** | **-** | **-** | **-** | **-** | **-** |
| Jiang 2020** [57] | 3 x 3mm2 | 3 x 3mm2 | **-** | **-** | **-** | 3 x 3mm2 | **-** | **-** | **-** | 3 x 3mm2 | **-** | 3 x 3mm2 | **-** |
| Farci 2020* [52] | 6 x 6mm2 | 6 x 6mm2 | 6 x 6mm2 | 6 x 6mm2 | 6 x 6mm2 | 6 x 6mm2 | 6 x 6mm2 | 6 x 6mm2 | 6 x 6mm2 | 6 x 6mm2 | **-** | **-** | **-** |
| Cordon 2020****** [53] | 6 x 6mm2 | 6 x 6mm2 | 6 x 6mm2 | 6 x 6mm2 | 6 x 6mm2 | **-** | **-** | **-** | **-** | 6 x 6mm2 | **-** | 6 x 6mm2 | **-** |
| Cennamo 2020* [54] | 6x6mm2 | 6x6mm2 | **-** | **-** | **-** | 6x6mm2 | **-** | **-** | **-** | 4.5x4.5mm2 | **-** | 4.5x4.5mm2 | **-** |
| Spain 2018*** [48] | 3x3x3 mm3 | **-** | **-** | **-** | **-** | **-** | **-** | **-** | **-** | 3x3x3 mm3 | **-** | **-** | **-** |
| Lanzillo 2018* [31] | 6x6 mm2 | **-** | 6x6 mm2 | 6x6 mm2 | **-** | **-** | **-** | **-** | **-** | **-** | **-** | **-** | **-** |
| Wang 2014*** [46] | 3x3x3 mm3 | **-** | **-** | 3x3x3 mm3 | **-** | **-** | **-** | 3x3x3 mm3 | **-** | 3x3x3 mm3 | **-** | **-** | **-** |

* Indicates studies using an Optovue machine, ** indicates studies using a Zeiss machine. *** indicates studies using a prototype Axsun SS-OCT machine, **** indicates studies using a Nidek machine, ***** indicates studies using a Heidelberg machine, and ****** indicates studies using a Topcon machine

–: Data not reported.
