# Supplementary material for: Optical coherence tomography angiography measurements in multiple sclerosis: a systematic review and meta-analysis
Source: J Neuroinflammation. 2023 Mar 27;20:85. doi: 10.1186/s12974-023-02763-4 (PMC10041805; doi:10.1186/s12974-023-02763-4)
Supplement: Supplementary file 3 — Additional file 3: Table S3. Exclusion criteria and additional information of included studies. [file 12974_2023_2763_MOESM3_ESM.docx]

| **First author/Year** | **Ophthalmological exclusion criteria** | **Eyes** | **Pupil dilation** | **Health conditions exclusion criteria** | **Adjusted for** |
| --- | --- | --- | --- | --- | --- |
| Aly 2022 [56] | Patients with substantial eye disease that may affect the integrity of the retinal architecture or vasculature (macular degeneration, retinal tumor, retinal detachment, vascular occlusions, history of eye surgery), refractory errors > 6 diopters (internal standard), or a relapse within 90 days before study enrolment. | Mixed | - | Individuals with MOGAD | Age/Sex |
| Ava 2022 [55] | Glaucoma and suspicion of glaucoma, visual acuity below 20/200, refractive error > ±3D, intraocular infection, hypertensive or diabetic retinopathy, ischemic optic neuritis, and retinal vascular occlusion, refractive surgery history, and a fundus that could not be clearly visualized (corneal opacification, cataract, etc.). | Mixed | - | Oral steroid treatment within 30 days/ MS exacerbation within 30 days | Age/Sex |
| Jesus 2021 [51] | Congenital eye disorders; myopia greater than 6 diopters; history of ocular surgery; presence of significant lens opacities or any macular disease; previous diagnosis of glaucoma; evidence of vitreoretinal disease, uveitis and diabetic retinopathy/In the control group, all patients had to have a normal anterior segment ophthalmic examination, a BCVA of 0.1 LogMar or better, no history of intraocular surgery, or any retinal pathological feature. | One | - | Did not present with any recent relapses (last 30 days) | Age/Sex/Axial length |
| Khader 2021 [36] | Patients with any media opacity as corneal opacity or dense cataract that interferes with the quality of imaging, patients with any other retinal disease as diabetic retinopathy, high myopia, retinal degeneration, and dystrophy, and patients diagnosed with any other causes of optic neuropathy like glaucoma, ischemic, and compressive optic neuropathy were excluded from the present study/patients with acute attacks of ON. Eyes with refraction with spherical equivalent less than - 6.00 Diopters and more than +2.00 Diopters. | One | - | Patients with other types of MS as progressive MS or patients with other demyelinating diseases like neuromyelitis optica or acute disseminating encephalomyelitis | Age/Sex/Refraction |
| Lee 2021 [50] | Patients with clinical features compatible with ischemic optic neuropathy (e.g., age 40 years or older, vasculopathic risk factors, no retrobulbar optic nerve enhancement on the pre- and post-contrast MRI, and no response to steroids)./Patients who were within six months of an ON attack on the date of the OCT and OCT-A examinations /Eyes with discontinuities in the blood vessels in the OCT-A image. | One in HC (but cases was mixed) | - | Less than 20 years of age or older than 70 years of age, history of any form of neurological impairment or diagnosis of a neurologic disorder such as NMOSD or MS prior to the first ON episode, history of systemic vasculitis/history or presence of malignancy or ocular pathology that could affect visual function including retinal disease, and optic neuropathies other than optic neuritis such as glaucoma and compressive optic neuropathies. | Age/Sex/Spherical equivalent refractive errors |
| Rogaczewska 2021 [43] | Myopia greater than 6 diopters, optic disc drusen, hypertensive or diabetic retinopathy, glaucoma, history of uveitis, or eye surgery | Both (but some were excluded due to low image quality) | - | Age < 18 years, ON attack within 6 months prior to examination, less than 2 years of disease duration for MS patients | Age/Sex |
| Liu 2021 [29] | Ocular diseases, including glaucoma, cataract, myopia  (<-6 diopter), or hyperopia (>6 diopter), or eye surgeries | Both (but some were excluded due to low image quality) | - | Diagnosis of other systemic diseases, including hypertension or diabetes/age <18 years; and inability to provide informed consent. | Age/Sex/inter-eye correlation in each participant |
| Yilmaz 2020 [45] | Presence of other ophthalmological pathologies that would confound the assessment results (glaucoma, diabetic or hypertensive retinal diseases, amblyopia, age-related macular degeneration,etc.), previous intraocular surgery, inability to cooperate OCT scanning, refractive error greater than ± 6 dioptres | Both | Yes | Corticosteroids usage, or MS exacerbation in the past 30 days. | - |
| Ulusoy 2020 [47] | Significant media opacities, a refractive error >=±6 diopter, history of ocular trauma, other systemic diseases, any other retinal vascular diseases, any other retinal disorders, and optic nerve diseases especially glaucoma | Both | - | used corticosteroids within 30 days of the start of the study, MS exacerbation in the 60 days prior to enrollment, and patients with any systemic vascular disease (e.g. hypertension, diabetes mellitus), hyperlipidemia and rheumatologic  disease which could affect the vascular system/patients, who only had ON symptoms associated with MS | Age/Sex |
| Murphy 2020 [22] | Ophthalmological disorders, eyes with prior ocular surgery or trauma or acute ON within the preceding 6 months, refractive errors of ±6 diopters, | Both(but some were excluded due to low image quality) | No | Potentially confounding neurological disorders, moderate to poorly controlled hypertension or diabetes mellitus | Age/Sex/Disease Duration/ON history/within-participant inter-eye correlations/ |
| Murphy 2020 [49] | (1) the diagnosis of ON was uncertain, (2) any elements of  the history or ophthalmologic examination were suggestive of an alternative diagnosis (e.g., ischemic optic neuropathy, retinal artery, or vein occlusion), (3) the laterality (i.e., right vs. left) of an ON episode was unclear, (4) the history included multiple episodes of ON, or (5) the history included bilateral ON./ophthalmological co-morbidities (e.g., glaucoma, macular degeneration, history of any other relevant retinal pathology such as retinal vascular occlusion or retinal detachment), prior Eye trauma or ocular surgery, refractive errors of >6 or <-6 dioptres. | Both | - | Primary progressive MS,secondary progressive MS (SPMS), seropositivity for myelin oligodendrocyte glycoprotein IgG (MOG-IgG), or seropositivity for aquaporin-4 IgG (AQP4-IgG), relevant known neurological co-morbidities/poorly-controlled hypertension, or poorly-controlled diabetes mellitus. | Age, sex, hypertension, diabetes |
| Jiang 2020 [57] | Subjects with ophthalmologic , such as macular edema, macular degeneration, glaucoma, diabetic and hypertensive retinopathy, or a refractive error greater than +-6 diopters were excluded from the study | Both(but some were excluded due to low image quality) | - | Neurologic disorders (other than MS) | Age/Sex |
| Farci 2020 [52] | Patients with macular degeneration, glaucoma, diabetic  retinopathy, or vitreomacular diseases | Both(but some were excluded due to low image quality) | - | - | Age/Sex |
| Cordon 2020 [53] | Elegant ocular (epiretinal membranes, glaucoma, age related macular disease etc)/Subjects with visual acuity < 0.4 decimal (6/15 on the Snellen chart), intraocular pressure > 20 mmHg, refractive errors greater than 5 diopters of spherical equivalent refraction or 3 diopters of astigmatism/prior ocular surgery | Single | - | Systemic disease related with retinal vascular density such as diabetes mellitus and arterial hypertension, active MS flare (of any neurological deficit) in the 6 months prior to enrolment in the study | Age/Sex/IOP |
| Cennamo 2020 [54] | Clinically relevant lens opacities/myopia >6 diopters/history of intraocular surgery, vitreoretinal, and  retinal vascular diseases, uveitis, congenital eye disorders/history of ON | Both | - | Relapse and/or corticosteroid use in the previous month-the presence of systemic vascular diseases (high blood pressure, diabetes, and heart diseases) | age, sex, best-corrected visual acuity, and visual field |
| Spain 2018 [48] | Evidence that could confound OCT interpretation such as intraocular  pressure >21 mm Hg, inability to maintain visual  fixation, and refractive errors greater than +3 or  -7 diopters in an ophthalmological exam within the last year | Both | - | Intravenous or oral steroids in the prior 30 days, MS exacerbation in the prior 60 days | age,sex,presence of vascular risk factors,inter eye correlation |
| Lanzillo 2018 [31] | The presence of congenital eye disorders; myopia greater than 6 diopters; history of ocular surgery; presence of significant lens opacities or any macular disease; previous diagnosis of glaucoma; evidence of vitreoretinal disease, uveitis and diabetic retinopathy; | Both | *-* | The presence of vascular comorbidities in particular: hypertension, statin therapy, diabetes and heart diseases. | Age |
| Wang 2014 [46] | Evidence on ophthalmological exam within the last  year of other ocular diseases or pathology that would confound the assessment (eg, glaucoma, diabetic or hypertensive retinal disease, amblyopia, etc.), previous intraocular surgery except for uncomplicated cataract extraction with posterior chamber intraocular lens implantation, inability to cooperate with OCT scanning, or refractive error greater than +3 or -7 dioptres | One | - | Intravenous or oral steroids in the prior 30 days, MS exacerbation in the prior 60 days | IOP,visual acuity,EDSS,EDMUS |

–: Data not reported.

Acronyms used in the table: OCT-A: Optical coherence tomography angiography/ RRMS: Relapsing remitting MS/ MSON: Multiple sclerosis with Optic neuritis/ MSNON: Multiple sclerosis without Optic neuritis/ HC: Healthy controls/MS: Multiple sclerosis/ VD: Vessel area density / VVD: Volumetric vessel density/RPC: Radial peripapillary capillary/ PD: Vessel perfusion density/ VVD: Volumetric vessel density/ IED: Inter eye difference/ Inf: Inferior quadrant/ Temp: Temporal quadrant/ Sup: Superior quadrant/ Nasal: Nasal quadrant/ MOGAD: MOG-antibody associated disease / BCVA: Best corrected visual acuity / ONH: Optic nerve head/ MRI: Magnetic resonance imaging/ ON: Optic neuritis/ IOP: Intra ocular pressure/ EDSS: Expanded Disability Status Scale score / EMDUS: physician-rated European Database for Multiple Sclerosis grading score
